# Dose-response relationship between very vigorous physical activity and cardiovascular health assessed by heart rate variability in adults: Cross-sectional results from the EPIMOV study

**DOI:** 10.1371/journal.pone.0210216

**Published:** 2019-01-31

**Authors:** Thiago Luís Wanderley de Sousa, Thatiane Lopes Valentim di Paschoale Ostoli, Evandro Fornias Sperandio, Rodolfo Leite Arantes, Antônio Ricardo de Toledo Gagliardi, Marcello Romiti, Rodrigo Pereira da Silva, Victor Zuniga Dourado

**Affiliations:** 1 Department of Human Movement Sciences, Federal University of São Paulo (UNIFESP), Santos, São Paulo, Brazil; 2 Angiocorpore Institute of Cardiovascular Medicne, Santos, São Paulo, Brazil; 3 Lown Scholars Program–Harvard T.H. Chan School of Public Health, Boston, Massachusetts, United States of America; University of Bourgogne France Comté, FRANCE

## Abstract

The minimum amount of physical activity needed to obtain health benefits has been widely determined. Unlikely, the impact of extreme amounts of very vigorous physical activity (VVPA, ≥ 8 metabolic equivalents) to the heart remains controversial. We aimed to evaluate the dose-response relationship between VVPA and heart rate variability (HRV) in adults. We selected 1040 asymptomatic individuals (60% women, 42 ± 15 years, 28 ± 6 kg/m2) from the Epidemiology and Human Movement Study (EPIMOV). Participants remained in the supine position for 10 min, and we selected an intermediate 5-min window for HRV analysis. The standard deviation of the RR intervals, root mean square of RR intervals, successive RR intervals that differ > 50 ms, powers of the low-and high-frequency bands and Poincaré plot standard deviations were quantified. Participants used a triaxial accelerometer (Actigraph GT3x+) above the dominant hip for 4–7 consecutive days for quantifying their physical activity. We also evaluated the maximum oxygen uptake ($\dot{V}O_{2\max}$) during an exercise test. We stratified participants into five groups according to the VVPA in min/week (group 1, ≤ 1.50; 2, 1.51–3.16; 3, 3.17–3.54; 4, 3.55–20.75; and 5, > 20.75). The linear trends of the HRV through the quintiles of VVPA were investigated. We used logarithmic transformations to compare the five groups adjusted for age, sex, cardiovascular risk, and $\dot{V}O_{2\max}$. We found a better HRV with increased VVPA for all HRV indices studied (p trend < 0.05). However, group 5 did not differ from group 4 (p > 0.05) for none of the indices. We conclude that there is an incremental benefit of VVPA on HRV of asymptomatic adults. Since we found neither additional benefits nor the harmful impact of amounts of VVPA as high as 22 min/week on HRV, our results should not discourage asymptomatic adults to perform VVPA.

## Introduction

Physical activity is defined as any body movement produced by skeletal muscles that result in energy expenditure higher than basal energy expenditure [1]. The minimum amount of physical activity related to beneficial cardiovascular adaptations is from moderate to vigorous intensity exercises lasting ≥ 30 minutes per day, 5 days per week, or vigorous exercises lasting ≥ 25 minutes per day, 3 days a week, or a combination of both to achieve a total energy expenditure ≥ 500–1000 metabolic equivalents (MET)/min/week [2].

However, the relationship between excessive amounts and intensities of physical activity and cardiovascular health is not well elucidated. Although the results are controversial, Lavie et al. [3] have observed in a systematic review of the literature that the achievement of an excessive amount of exercise, defined as > 60–90 minutes per session, is associated with a risk of cardiotoxicity and therefore with deleterious effects on cardiovascular health.

Heart rate variability (HRV) is a simple and widely used measure for cardiovascular health. Heart rate variability is the phenomenon of the difference between RR intervals of consecutive heartbeats and indirectly reflects the dynamics between the sympathetic and parasympathetic mechanisms of heart rate regulation [4]. The decline in HRV is related to increased mortality after acute myocardial infarction [5], congestive heart failure [6], cancer [7] and diabetes mellitus [8]. It has been observed that the combination of HRV indices may identify hypertensive patients at high risk to develop future vascular events [9]. It is also associated with overtraining syndrome [10].

There is evidence that physical activity is associated with better HRV [11]. However, how much extreme amounts of very vigorous physical activity (VVPA) may result in additional benefits in HRV remains unknown. VVPA can be obtained directly through the use of triaxial accelerometers and is defined by physical activities performed with energy expenditure equal to or above 8 METs.

In the present study, we hypothesized that excessive amounts of VVPA are not related to additional benefits on HRV. Therefore, we aimed to evaluate the dose-response relationship between the amount of VVPA and cardiovascular health assessed by HRV in asymptomatic adults.

## Materials and methods

### Participants and design

**Fig 1:**
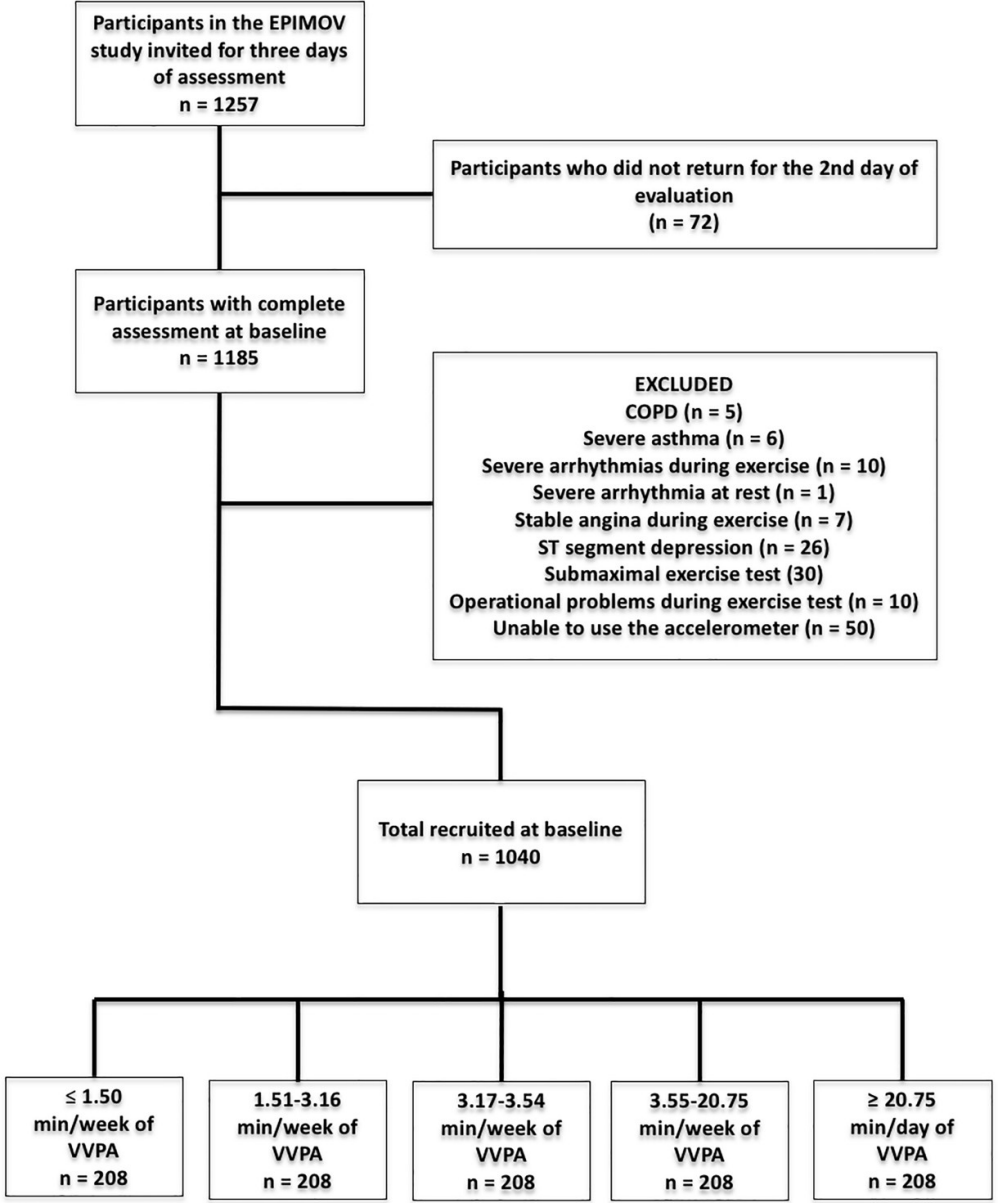
Flowchart of the study. EPIMOV: The Epidemiology and Human Movement Study. COPD: chronic obstructive pulmonary disease; VVPA: very vigorous physical activity.

We conducted a cross-sectional study with a convenience sample. We enrolled 1040 individuals who participated in the baseline evaluation of the Epidemiology and Human Movement Study (EPIMOV Study) as in Fig 1. The EPIMOV study is a prospective cohort study that aims to determine the association between low levels of physical activity and fitness and the occurrence of chronic hypokinetic diseases, mainly cardiorespiratory and locomotor diseases. Individuals of both sexes aged between 18 and 80 years old, without previously diagnosed pulmonary, cardiovascular, metabolic, musculoskeletal and neuromuscular diseases were recruited through social networks, universities in the region, magazines and local newspapers.

Exclusion criteria were the use of gaiters, recent respiratory infections, unstable angina at baseline, stable angina, arrhythmias or electrocardiographic abnormalities during exercise testing and the refusal of participants. We also excluded participants who demonstrated spirometric indices suggestive of pulmonary diseases.

Participants were informed about the possible risks and discomforts of the evaluations and signed an Informed Consent Term. The Human Research Ethics Committee of the Federal University of São Paulo (number 186,796) approved the present study.

### Study measurements of the EPIMOV

At each scheduled assessment period of the EPIMOV, study measurements are carried out over the course of two visits spaced seven days apart. In the first visit, participants undergo general health screening, anthropometrics, spirometry and cardiopulmonary exercise testing (Quark PFT, COSMED, Italy). At the end of the first assessment, we instruct participants about using the triaxial accelerometer for the subsequent seven days (Actigraph GT3x +, MTI, Pensacola, FL, USA). In the second visit, they return the accelerometer, answer the international physical activity questionnaire (IPAQ), and then undergo assessments of heart rate variability (V800, POLAR, Finland), body composition (bioelectrical impedance) (310e, Biodynamics, United States), isokinetic muscle function (System 4, Biodex, United States) and postural balance (force platform) (BIOMEC400, EMGSystem, Brazil). At the end of the second visit, they undergo a six-minute walk test. Participants are also asked to contribute a blood sample between two visits. In the present study, we analyzed the results of the following assessments:

### Spirometry

The forced vital capacity maneuver was carried out using a calibrated spirometer (Quark PFT, COSMED, Italy). Since the EPIMOV Study was designed to investigate mainly obstructive pulmonary disease, we carried out the spirometry before and 15 minutes after inhalation of 400 micrograms of salbutamol [12] in the presence of forced vital capacity (FVC) to forced expiratory volume in 1 s (FEV1) ratio below 70%.

### Cardiorespiratory fitness

Participants performed a symptom-limited cardiopulmonary exercise test under a treadmill ramp protocol (ATL, Inbrasport, Brazil). After 3 min at rest, we increased velocity and inclination according to the estimated maximum oxygen uptake ($\dot{V}O_{2\max}$), with the aim of finishing the test in about 10 minutes.

Cardiovascular, ventilatory and metabolic variables were collected breath by breath using a gas analyzer (Quark PFT, Cosmed, Italy). We measured oxygen uptake ($\dot{V}O_{2}$), carbon dioxide output ($\dot{V}CO_{2}$), and minute ventilation throughout the test. Heart rate (HR) was monitored continuously during the test using a 12-lead electrocardiogram (C12x, COSMED, Italy). The data were filtered every 15 seconds for further analysis. The peak $\dot{V}O_{2}$ was considered as the mean value in the last 15 seconds at the peak of the incremental exercise and is presented in absolute (mL/min), corrected for body weight (mL/min/kg) and as percentage of reference values (% pred.) [13].

We discontinued the CPET in cases of potentially dangerous arrhythmias, signs or symptoms suggestive of myocardial ischemia or patient's request. These tests were considered ineffective when did not reach 85% of the predicted maximum HR for age and/or $\dot{V}CO_{2}/\dot{V}O_{2}$ ≥ 1.0 [14].

### Physical activity level

We evaluated the level of physical activity through the use of triaxial accelerometers (Actigraph GT3x +, MTI, Pensacola, FL, USA). The volunteers wore the device at the waist above the dominant hip for at least ten waking hours a day for a week. They were also instructed to remove the device for water-related activities, contact sports, and nighttime sleep. We defined the wearing-time at 24 h minus non-wearing time. We considered non-wearing time as an interval of zero counts for 60 or more minutes.

The thresholds for physical activity intensity were as follows: very light (100–759 counts per minute); light (760–1951 counts per minute), moderate (1952–5724 counts per minute); vigorous (5725–9498 counts per minute) and very vigorous (≥ 9499 counts per minute). The VVPA threshold represented an intensity equal to or above 8 METs. The sedentary time was considered based on the periods with less than 100 counts per minute, representing < 1.5 MET [15].

### Anthropometric and body composition

We measured height and body weight with a scale and a stadiometer, and then the body mass index (BMI) was calculated. Body composition was determined by bioelectrical impedance (310E BIODYNAMICS, Detroit, USA), following the procedures described by Kyle et al. [16]. Then, we calculated lean and fat body masses using a regression equation developed for healthy subjects [17].

### Heart rate variability

The volunteers underwent resting heart rate evaluation in the supine position for 10 minutes, using a heart rate monitor (POLAR RS800cx). Participants were instructed not to ingest stimulant substances (e.g., beverages containing caffeine), to smoke, and to perform physical activities before evaluation. For analysis purposes, we analyzed only the intermediate 5-minute window. Data were transferred to a computer using the compatible software (Polar ProTrainer 5, Polar). After visual inspection, we analyzed the data using a free software (Kubios HRV, version 2.2). If an RR interval differed from the locale average more than a specific threshold value, the interval was identified as an artifact and was marked for correction. We have chosen the medium artifact correction which means that any RR interval that was larger/smaller than 0.25 s compared to the locale average was corrected by replacing the identified artefact with interpolated values using a cubic spline interpolation. We adjusted this threshold with mean heart rate.

We quantified in the time domain the standard deviation of the RR intervals. The linear indices obtained in the time domain were as follows: the mean RR interval, the root mean square of successive differences between adjacent normal RR intervals, the standard deviation of the RR intervals, the number of adjacent normal RR intervals differing by > 50 ms, and the proportion of adjacent normal RR intervals differing by > 50 ms.

In the frequency domain, we evaluated the powers of the high- (HF, 0.15–0.40 Hz), low- (LF, 0.04–0.15 Hz) and very low (VLF, 0–0.04 Hz) frequency bands. We expressed HF, LF and VLF in absolute and normalized values. We used the fast Fourier transform (FFT) in which the RR intervals were resampled in the time (4 Hz), and the frequency spectrum was divided into 1,024 points [18]. We also calculated the LF/HF ratio.

Finally, we quantified Poincaré plot standard deviations, both along and perpendicular to the regression line [4]. We analyzed the Poincare plot in the Cartesian scatergram in which each RR interval was correlated with the preceding interval. The index SD1, which represents the dispersion of points perpendicular to the line of identity and corresponds to the instantaneous beat-to-beat variability, and the index SD2, which represents the standard deviation of long-term continuous RR intervals, were calculated in this method.

All participants remained at rest for 5 min before the test and were instructed to breathe normally and avoid speaking during the test.

### Statistical analysis

Statistical analysis was performed using SPSS software, version 23 (IBM, USA). The volunteers were divided into 5 groups according to the VVPA quintiles in min/week (group 1, ≤ 1.50; 2, 1.51–3.16; 3, 3.17–3.54; 4, 3.55–20.75; and 5, > 20.75). Initially, we performed analyses of variance (ANOVA) to investigate differences in demographics, anthropometrics, body composition, physical activity, physical fitness and cardiovascular risk factors among VVPA quintiles. We evaluated the linear trend of HRV through quintiles of the VVPA (p for trend). Before the multivariate analysis of the data, we evaluated the bivariate correlations between the HRV indexes and the covariables studied using the Pearson or Spearman correlation coefficients, as well as biserial correlations (e.g., for categorical dichotomous variables). We then used logarithmic transformations to normalize the data so that we could compare the five groups in multivariate analyses of covariance (ANCOVA). In these models, we considered the HRV indices as the outcomes and the VVPA quintiles as the main factor. We adjusted the models for the main covariates, i.e., age, sex, schooling, cardiovascular risk and $\dot{V}O_{2\max}$. Post-hoc comparisons and Bonferroni correction were used to identify between group differences.

To increase the statistical power of the multivariate models, we calculated the Framingham cardiovascular risk score (CVRS), including age, sex, body mass index, hypertension, and its treatment, diabetes, and smoking [19]. We did a sensitivity analysis and, due to the strength of the correlation, we chose the CVRS as a covariate instead of inserting the cardiovascular risk factors. The CVRS was calculated as a percentage as recommended [19]. Both the peak $\dot{V}O_{2}$ and the CVRS were included in the multivariate models dichotomized in their medians.

We also performed a series of additional multivariate analyzes including moderate physical activity as a covariate. We chose to put the amount of moderate physical activity in the model, dichotomized in its median, instead of the MVPA, since MVPA already mostly compose VVPA. Our missing data were due to non-response and represented less than 20% of the total. For this reason, we evaluate the data without multiple imputations. We set the probability of alpha error at 5% for all tests.

## Results

The present study evaluated 1040 individuals without symptomatic chronic diseases. The individuals were mostly middle-aged women, with BMI indicating overweight and with the proportion of cardiovascular risk factors compatible with the Brazilian population. Participants in fourth and fifth VVPA quintiles were predominantly men, on average younger and with better anthropometric and body composition indices. Also, they presented lower proportions of risk factors for cardiovascular diseases (Table 1).

**Table 1:** General characteristics of the studied sample according to the quintiles of very vigorous physical activity (n = 1040).

Variables	Quintile 1 (n = 208)	Quintile 2 (n = 208)	Quintile 3 (n = 208)	Quintile 4 (n = 208)	Quintile 5 (n = 208)
**Age (years)**	50 ± 14^345^	45 ± 14^345^	45 ±15^45^	38 ± 14	36 ±13
**Females (%)**	75.6^45^	67.0^45^	65.3^5^	57.6	43.9
**BMI (Kg/m^2^)**	29 ± 6^5^	30 ± 6^145^	29 ± 6^45^	27 ± 5	25 ± 4
**Fat body mass (% of total)**	34 ± 8^45^	34 ± 8^45^	32 ± 10^45^	28 ± 9	24 ± 8
**Systolic blood pressure (mmHg)**	131 ± 14^5^	132 ± 18^5^	129 ± 13	129 ± 12	125 ± 10
**Diastolic blood pressure (mmHg)**	82 ± 8^5^	82 ± 9^5^	82 ± 8^5^	82 ± 8^5^	79 ± 6
**Arterial hypertension (%)**	23.5^45^	26.7^45^	18.2^5^	13.9	7.2
**Diabetes (%)**	15.0^45^	13.6^5^	9.4	5.5	3.6
**Dyslipidemia (%)**	33.3^45^	29.5^5^	31.2^5^	19.4	13.3
**Obesity (%)**	44.1^45^	44.9^45^	40.6^5^	27.3^5^	12.7
**Current smoking (%)**	15.0	13.1	14.7	8.5	9.6

Quintiles of very vigorous physical activity in min/week (group 1, ≤ 1.50; 2, 1.51–3.16; 3, 3.17–3.54; 4, 3.55–20.75; and 5, > 20.75).

Continuous variables are presented as the mean ± standard deviation and categorical variables with %.

The superscript numbers represent a statistically significant difference compared to the quintile numbers (p <0.05).

BMI: body mass index.

Participants in groups 4 and 5 of VVPA also presented a better level of physical activity and fitness. There was no statistically significant difference between groups in sedentary time or light-intensity physical activity (Table 2).

**Table 2:** Physical activity and fitness according to the quintiles of very vigorous physical activity.

Variables	Quintile 1 (n = 208)	Quintile 2 (n = 208)	Quintile 3 (n = 208)	Quintile 4 (n = 208)	Quintile 5 (n = 208)
**Time spent in physical activity intensities**					
Light (min/week)	1054 ± 353	1186 ± 483	1301 ± 415	1205 ± 430	1158 ± 357^3^
Moderate (min/week)	168 ± 99	225 ± 120	258 ± 109	302 ± 156^123^	321 ± 134^123^
Vigorous (min/week)	0.8 ± 0.4	2.1 ± 0.5	4.3 ± 0.9	10.6 ± 3.9^123^	54.5 ± 49.7^1234^
Very vigorous (min/week)	0.03 ± 0.07	0.11 ± 0.15	0.29 ± 0.48	1.37 ± 2.37	10.07 ± 20.79^1234^
MVPA (min/week)	169 ± 99	227 ± 120	263 ± 109^12^	314 ± 156^123^	385 ± 149^1234^
MVPA (min/day)	29.9 ± 16.8	38.0 ± 16.7	43.0 ± 15.3^12^	51.4 ± 21.0^123^	61.9 ± 21.3^1234^
Sedentary (min/week)	3556 ± 961	3733 ± 1302	3743 ± 955	3698 ± 871	3884 ± 847^1^
Cardiorespiratory fitness*					
$\dot{V}O_{2\max}$ (mL/min/kg)	26.5 ± 8.4	27.5 ± 9.2	30.7 ± 10.0^12^	37.9 ± 9.8^123^	41.9 ± 9.1^1234^
$\dot{V}O_{2\max}$ (% pred.)	95 ± 16	95 ± 15	99 ± 15	106 ± 16^123^	118 ± 24^1234^
Mean HR (bpm)	70 ± 10	69 ± 12	69 ± 10	67 ± 10	62 ± 12^1234^
Mean RR (ms)	876 ± 125	885 ± 145	885 ± 126	914 ± 134	988 ± 207^1234^

Quintiles of very vigorous physical activity in min/week (group 1, ≤ 1.50; 2, 1.51–3.16; 3, 3.17–3.54; 4, 3.55–20.75; and 5, > 20.75).

MVPA: moderate to vigorous physical activity; $\dot{V}O_{2}$: oxygen uptake; HR: heart rate; RR: RR interval

The superscript numbers represent a statistically significant difference compared to the quintile numbers (p <0.05).

*treadmill cardiopulmonary exercise testing

The results are expressed as the mean ± standard deviation.

**Fig 2:**
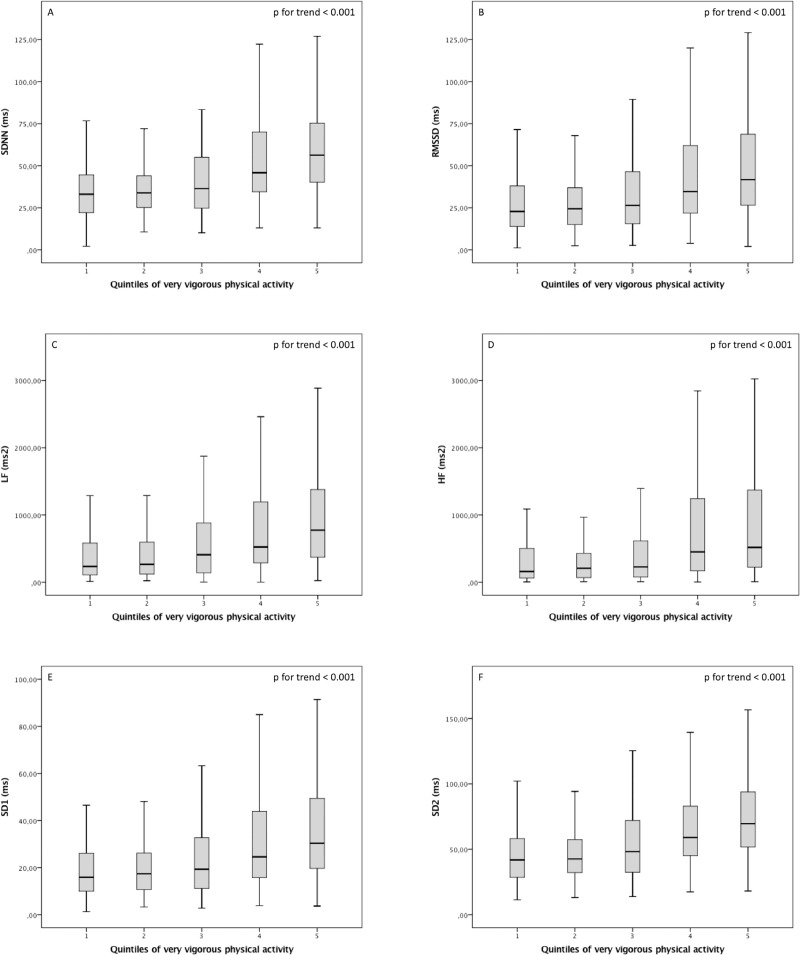
The linear trend of heart rate variability indices across the very vigorous physical activity quintiles in min/week: group 1, ≤ 1.50; 2, 1.51–3.16; 3, 3.17–3.54; 4, 3.55–20.75; and 5, > 20.75. SDNN (A): the standard deviation of the RR intervals; RMSSD (B): root mean square of RR intervals; LF (C) and HF (D): respectively powers of the low- and high-frequency bands; SD1 (E) and SD2 (F): respectively perpendicular and along with regression line of the Poincaré plot standard deviations.

All variables analyzed showed a significant linear trend through the VVPA quintiles, indicating a significant positive correlation between VVPA and better HRV (p for trend < 0.001) (Fig 2).

**Fig 3:**
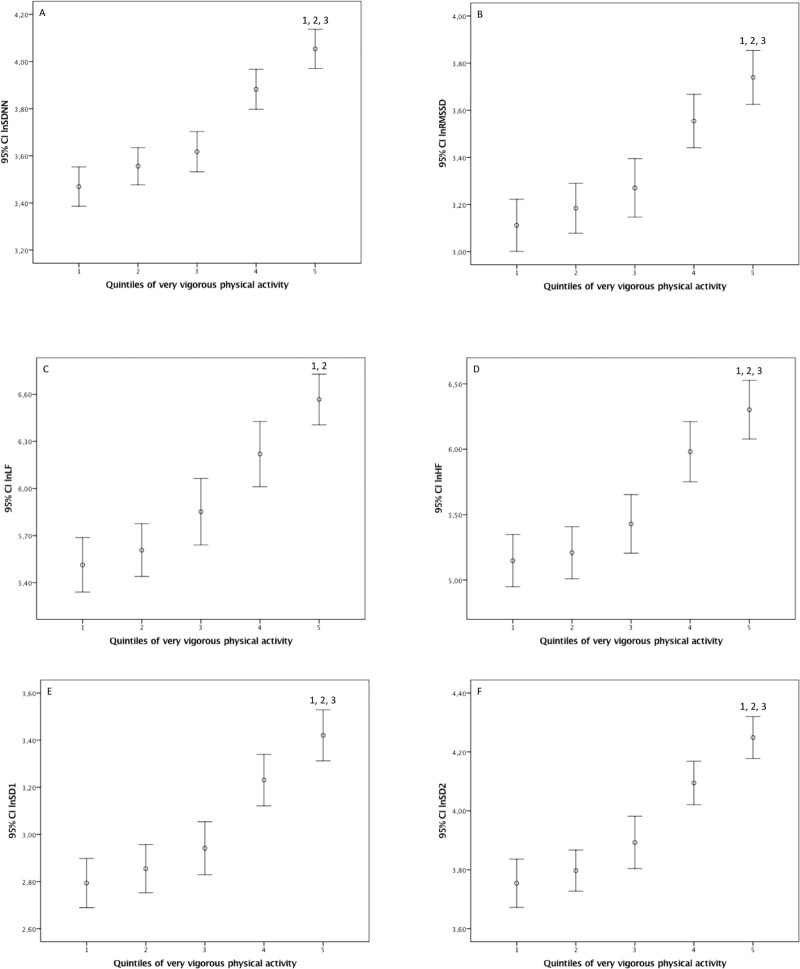
Results of the multivariate analysis of covariance (ANCOVA) with the differences between the very vigorous physical activity quintiles in min/week for the heart rate variability normalized utilizing logarithmic transformations (group 1, ≤ 1.50; 2, 1.51–3.16; 3, 3.17–3.54; 4, 3.55–20.75; and 5, > 20.75). SDNN (A): the standard deviation of the RR intervals; RMSSD (B): root mean square of RR intervals; LF (C) and HF (D): respectively powers of the low- and high-frequency bands; SD1 (E) and SD2 (F): respectively perpendicular and along with regression line of the Poincaré plot standard deviations.

However, after multivariate analysis adjusted for the main covariates, we did not observe significant differences between quintiles 4 and 5 for any of the HRV indices studied, indicating no additional benefit in HRV from 20.75 min/week of VVPA (Fig 3). Also, in a second set of multivariate ANCOVA adjusted additionally for moderate physical activity, all the results but LF presented exactly the same results. As for the LF, we found difference only between quintile 5 and 2.

## Discussion

We have shown in the present study that excessive amounts of very vigorous physical activity, i.e., equal to or greater than 8 MET, are associated neither with healthier nor with worse heart rate variability indices. We, therefore, suggest a maximum threshold from which VVPA is no longer able to produce benefits in autonomic modulation in adults. To our knowledge, this is the first study to evaluate physical activity directly and its association with autonomic modulation in this context.

The main finding of the present study was the plateau found in the benefits of very vigorous physical activity in HRV. We observed a positive correlation between the increase in the amount of VVPA and better HRV, indicating incremental benefits in the autonomic modulation with the increase of VVPA. However, our results suggest that there are no additional benefits in HRV from about 21 min/week of VVPA. The previous literature suggests a "U" relationship between physical activity and mortality. Schnohr [20] in a prospective 12-year follow-up study showed that there was no significant difference in mortality between sedentary non-runners and individuals who ran in an extreme amount and intensity. In this study, the group performing the highest amount of physical activity was composed of participants who performed more than 4 hours per week of VVPA or competitive sports several times per week. Regarding intensity, this group would run at speeds equal to or greater than 7 miles/h, which represents 12 METs or more in terms of intensity. Our results suggest a plateau concerning the benefit of VVPA on HRV and are very similar to the group considered as moderate-intensity in the study of Schnohr et al. [20], i.e., between 6 and 12 MET, which showed a lower risk of mortality compared to non-runners, but no benefit over the light jogging group (up to 6 METs). Therefore, both studies suggest that 8 METs may be a threshold for cardiovascular benefits.

Our results additionally suggest that autonomic modulation may play an essential role in explaining poorer cardiovascular health related to excessive amounts and intensities of physical activity. Good autonomic modulation is essential for maintaining heart rhythm. In fact, the worst HRV has been associated with a higher cardiovascular mortality, as well as with a higher incidence of sudden cardiac death [21]. Elliot et al. [22] showed that the ventricular arrhythmias observed in endurance athletes are mainly caused by cardiac morphological changes, mainly the reduction of right ventricular ejection fraction. Also, the U-shaped correlation was also identified, for example, between the long time excessive amount of vigorous endurance exercises and the occurrence of atrial fibrillation, with high-performance athletes presenting a 5-fold increased risk of developing this arrhythmia [23]. Another study has shown that more than 5 hours per week of very vigorous physical activity for people over the age of 30 substantially increases the risk of atrial fibrillation [24]. Our results reinforce the findings mentioned above since one of the central mechanisms that explain the higher prevalence of atrial fibrillation, both in athletes and in the general population, is the shortening of the atrial refractory period due to increased vagal tone [25, 26]. Finally, several arrhythmias were identified more frequently in athletes, as well as the occurrence of ventricular fibrosis (26). The clinical impact of these findings remains unclear. However, autonomic modulation may play an essential role in this context, which reinforces the interpretation of our results.

Our results may suggest > 20.75 min/week of VVPA as a maximum threshold for exercise prescription concerning HRV improvement, especially for individuals with a higher cardiovascular risk. However, we did not observe a deleterious impact of more massive quantities than this one in the HRV of the participants of the present study. Although our study is cross-sectional, our results reinforce a detailed pooled analysis of the dose-response relationship between leisure-time physical activity and adult mortality. Arem et al. [27] observed that individuals who performed less than the recommended for health benefits showed a reduction of 20% of the risk of death, with a progressive reduction of the risk for 1 to 2 or 2 to 3 times the recommended minimum. However, the authors observed a 39% lower mortality risk among those who performed 3–10 times the minimal recommendations. Above this threshold, there was a significant reduction in the risk of death, but not as strong (i.e., 32% lower). The same results were found with separate categories of moderate (3–6 METs) versus vigorous (≥ 6METs) physical activities. Specifically for cardiovascular mortality, however, the upper threshold was observed among those who reported 3–5 times the minimum recommendations. There was no additional benefit for those who reported 5–10 or above 10 times. Our study, therefore, reinforces the message that quantities and intensities of VVPA far above the recommended do not bring additional benefits, but also do not provoke deleterious effects for autonomic modulation as well as observed for mortality [27].

High-intensity physical exercises, especially of endurance, cause acute and chronic effects to the cardiovascular system. Significant increases in cardiac injury biomarkers such as troponin, creatine kinase and B-type natriuretic peptide were described in a considerable proportion in marathon runners both during and after the events [28–31]. In combination with a substantial increase in catecholamines, they alter preload and post-load and lead to diastolic dysfunction [32–34]. As a medium-term repercussion, individuals who perform excessive amounts of vigorous physical activity have a higher incidence of cardiac fibrosis [35]. As long-term results, the highest risk of atrial fibrillation, ventricular arrhythmias and even sudden cardiac death in endurance athletes with a long history of vigorous physical activities has been described [35]. A potential mechanism that may explain the association between excessive high-intensity exercise and the higher incidence of atrial, for example, is in changes in vagal and sympathetic activity in combination with increased atrial chamber and fibrosis [35, 36]. All of these changes related to excessive high-intensity physical exercise, especially altered autonomic balance, and may explain the lack of additional benefit in HRV in our participants of the last VVIG quintile.

It is important to note that HRV interpretation as representative of autonomic modulation should be made with caution, since more than a direct measure of autonomic activity, HRV is a local measure of modulation of an organ, i.e., of HR. Heart rate is not only under the control of the direct autonomic innervation at the sinoatrial node, but it can also be influenced by other aspects such as mechanical and hemodynamic effects, as well as by sympathoadrenal activation [37, 38]. Therefore, it is fundamental to consider HRV as an indirect measure of cardiac autonomic modulation. Therefore, we decided to present in the present study the main HRV indexes, which have shown for decades the most significant relationship with autonomic modulation and clinical applicability [18, 38], e.g., SDNN, RMSSD, HF, LF, SD1, and SD2.

We adjusted our multivariate models for age and sex. Older individuals had lower values of representative indices of the parasympathetic nervous system. With an increase in age, the HRV tends to decrease; this reduction can primarily be observed from the indices in the time domain [39, 40]. Women exhibited higher values of representative indices of the parasympathetic nervous system, whereas men showed sympathetic predominance in heart modulation, as previously described [40–43]. Also, HRV strongly declined with age and was consistently higher in women. These demographic factors together explained 17.4% to 21.9% in in a previous study, while adding lifestyle and psychosocial factors to the model additionally explained less than 0.50% of the variance in HRV [44]. Studies have suggested that female hormones, particularly β-estradiol, are involved in the facilitation of vagal activation in the heart [41]. Furthermore, the physical constitution of men would be one possible explanation for the sympathetic predominance; men have increased muscle sympathetic activity in addition to a greater number of sympathetic ganglia as compared to women [41].

Our results have practical implications for public health. Cardiovascular events related to vigorous physical activity have a possible mechanism in the autonomic imbalance. We observed that more than approximately 21 min/week of VVIG is not associated with additional HRV benefits and indirectly to the improvement in autonomic modulation. Vigorous physical activity transiently increases the risk of acute myocardial infarction by up to 50 times [45] and sudden cardiac death, particularly among physically inactive individuals with incipient or known coronary disease who engage in vigorous physical activities for which they are not accustomed [46]. Our participants were recruited from the general population and, although we evaluated highly active subjects, no professional athlete was included. Therefore, large amounts of VVIG in people of the general population, often with older age and cardiac comorbidities, should be prescribed with caution and after familiarization with moderate to vigorous regular physical activity to avoid adverse effects of physical exercise.

On the other hand, health professionals should not overestimate the risks of exercise because the benefits of habitual physical activity substantially outweigh the risks. It seems that one of the most important defenses against cardiovascular events related to exercise in adults is to maintain physical fitness through regular physical activity because a disproportionate number of exercise events occur in physically inactive individuals performing a vigorous physical activity for which they are not familiar [45]. Participant screening, knowledge of the symptoms that lead to cardiovascular events by the participant and the professional, as well as a progressive increase of exercise intensity are rational strategies to obtain the most significant benefits of VVIG and prevention of cardiovascular events [46].

The present study has limitations that must be considered. The cross-sectional design prevents us from interpreting a possible cause and effect relationship between VVPA and HRV over time. The EPIMOV study is a recent cohort study, and we intend to carry out such an analysis soon. Our convenience sampling may also have introduced a bias to our results. However, several demographics, anthropometric and cardiovascular risk characteristics in the present study are similar to those described for the Brazilian population.

## Conclusions

We may conclude that VVPA is associated with significant benefits in the autonomic modulation of asymptomatic adults. However, extremely high amounts of VVPA were not associated with additional HRV benefits. Since we found no deleterious impact of amounts of VVPA as high as 22 min/week on HRV, our results should not discourage asymptomatic adults to perform VVPA.
